# Steady‐state mobilization with on‐demand plerixafor after CD38 antibody‐based induction in multiple myeloma patients

**DOI:** 10.1111/trf.70165

**Published:** 2026-03-20

**Authors:** Maximilian Alexander Röhnert, Anna Seifert, Karolin Trautmann‐Grill, Christoph Röllig, Kristin Zimmer, Katharina Egger‐Heidrich, Marius Bill, Lisa Heberling, Freya Schulze, Matthias Blechschmidt, Malte von Bonin, Martin Bornhäuser, Kristina Hölig, Raphael Teipel

**Affiliations:** ^1^ Department of Medicine I, Faculty of Medicine and University Hospital Carl Gustav Carus TUD Dresden University of Technology Dresden Germany; ^2^ National Center for Tumor Diseases Dresden (NCT/UCC), a partnership between DKFZ, Carl Gustav Carus Faculty of Medicine and University Hospital Carl Gustav Carus TUD Dresden University of Technology, Helmholtz‐Zentrum Dresden‐Rossendorf (HZDR) Dresden Germany

## Abstract

**Background:**

High‐dose chemotherapy followed by autologous stem cell transplantation (ASCT) remains the standard of care for fit patients with newly diagnosed multiple myeloma (MM). The increasing use of CD38 antibody‐based quadruplet induction regimens such as daratumumab‐VTd (Dara‐VTd) has raised concerns regarding impaired stem cell mobilization.

**Study Design and Methods:**

We conducted a retrospective single‐center analysis comparing steady‐state stem cell mobilization after Dara‐VTd versus bortezomib–cyclophosphamide–dexamethasone (VCd) induction. Mobilization kinetics, plerixafor use, and CD34^+^ collection outcomes were evaluated. CD34^+^ counts prior to first apheresis were adjusted for plerixafor use (adjCD34^+^). Multivariate logistic regression was performed to identify predictors of mobilization success.

**Results:**

Among 153 patients, 85 received Dara‐VTd and 68 received VCd. Despite significantly deeper responses after Dara‐VTd (≥VGPR 81% vs. 42%; *p* < .01), these patients showed lower adjCD34^+^ counts prior to first apheresis (16 vs. 50/μL; *p* < .01) and required plerixafor more frequently (64% vs. 15%; *p* < .01). Nevertheless, cumulative CD34^+^ yields were comparable between Dara‐VTd and VCd (6.4 vs. 6.0 × 10^6^ CD34^+^ cells/kg; *p* = .15), and target yields were achieved in the majority of patients proceeding to apheresis (90% vs. 94%). Dara‐VTd induction, prior radiation, and high tumor burden were identified as independent negative predictors of mobilization success.

**Discussion:**

Although Dara‐VTd induction is associated with impaired mobilization kinetics, successful steady‐state mobilization remains feasible. On‐demand plerixafor use overcomes mobilization deficits, supporting this approach in patients receiving CD38‐based quadruplet induction therapy. Furthermore, follow‐up analysis of stem cell graft utilization demonstrates a high proportion of collected but unused stem cell grafts in both cohorts.

**Abbreviations**
adjCD34+: adjusted CD34+ count (prior to plerixafor)
ASCT: autologous stem cell transplantation
BM: bone marrow
bw: body weight
CXCR4: C‐X‐C chemokine receptor type 4
Dara‐VRd: daratumumab, bortezomib, lenalidomide, and dexamethasone
Dara‐VTd: daratumumab, bortezomib, thalidomide, and dexamethasone
FISH: fluorescence in situ hybridization
G‐CSF: granulocyte colony‐stimulating factor
IMiD: immunomodulatory drug
IMWG: International Myeloma Working Group
IQR: interquartile range
ISS: International Staging System
LPH1: first apheresis session
LPH2: second apheresis session
MM: multiple myeloma
MRD: minimal residual disease
OR: odds ratio
pB: peripheral blood
PBSC: peripheral blood stem cell
R‐ISS: Revised International Staging System
VCd: bortezomib, cyclophosphamide, and dexamethasone
VGPR: very good partial remission
WBC: white blood cells

## 1 INTRODUCTION

High‐dose chemotherapy followed by autologous stem cell transplantation (ASCT) remains a cornerstone of first‐line therapy for fit patients with newly diagnosed multiple myeloma (MM), despite the continuous evolution of novel agents in the treatment landscape of MM.^1^, ^2^ In recent years, the incorporation of CD38 monoclonal antibodies into proteasome inhibitor‐ and immunomodulatory drug (IMiD)‐based backbones has significantly improved depth and durability of response. Consequently, quadruplet induction therapy regimens, for example, Dara‐VTd (daratumumab, bortezomib, thalidomide, and dexamethasone) or Dara‐VRd (daratumumab, bortezomib, lenalidomide, and dexamethasone) have replaced former triplet regimens like VCd (bortezomib, cyclophosphamide, and dexamethasone) or VRd (bortezomib, lenalidomide, and dexamethasone). Induction therapy with a CD38 antibody‐based quadruplet combination for 4–6 cycles is nowadays considered as the standard of care before proceeding to high‐dose chemotherapy and ASCT.^3^, ^4^, ^5^

The collection of a sufficient autologous stem cell graft, defined as ≥2 × 10^6^ CD34^+^ cells/kg body weight is required for successful ASCT. Typically, at least two equivalent stem cell grafts are collected to ensure availability for a potential tandem or even later salvage ASCT. A steady‐state mobilization approach using granulocyte colony‐stimulating factor (G‐CSF) alone for five days is one of the commonly used methods for stem cell mobilization.^6^ Additionally, the CXCR4 antagonist plerixafor is used preemptively or as rescue therapy in patients showing insufficient mobilization.^7^, ^8^, ^9^ Several factors are known to impact the success of stem cell mobilization.^10^, ^11^, ^12^, ^13^, ^14^ In particular, the use of CD38 antibodies appears to have a detrimental effect on collection outcomes. Emerging data from both clinical trial and real‐world cohorts support this observation.^15^, ^16^, ^17^ Post hoc data from the MASTER and GRIFFIN‐trial reported of successful stem cell mobilization and collection outcomes, with an increased upfront or rescue use of plerixafor.^18^ Given the now widespread use of CD38 antibody‐containing induction regimens in clinical routine practice, this issue is becoming increasingly relevant. Therefore, we conducted a retrospective, monocentric study investigating differences in stem cell mobilization, collection, and engraftment kinetics between patients who received induction therapy with Dara‐VTd and those treated with VCd in a large patient cohort. Besides, we focused on the actual utilization of the collected stem cell grafts, thereby providing a relevant follow‐up after stem cell collection.

The present study therefore aimed to provide confirmatory real‐world evidence on the feasibility and effectiveness of a steady‐state mobilization strategy after CD38 antibody‐based quadruplet induction and to assess long‐term stem cell graft utilization.

## 2 PATIENTS AND METHODS

### 2.1 Study cohort and induction therapy

This retrospective single‐centre analysis included 153 ASCT‐eligible patients with newly diagnosed MM who started induction therapy with the intention to proceed to high‐dose chemotherapy with Melphalan followed by ASCT between July 2017 and October 2023. Patients received the respective standard‐of‐care induction regimen in Germany at the time of diagnosis, either bortezomib, cyclophosphamide, and dexamethasone (VCd) or daratumumab, bortezomib, thalidomide, and dexamethasone (Dara‐VTd). The choice of induction regimen was primarily determined by the EMA approval of Dara‐VTd in January 2020 and the subsequent change in first‐line treatment standards. All patients received 3–7 cycles of induction therapy (median 4 cycles in both groups).

### 2.2 Stem cell mobilization and collections

All patients initially underwent steady‐state mobilization according to local practice guidelines. In case of mobilization failure or inadequate stem cell yield despite the use of plerixafor, a second mobilization attempt was performed at a later time point using a cyclophosphamide‐based chemotherapy regimen in combination with G‐CSF.

For steady‐state mobilization, G‐CSF alone (mainly lenograstim) was used at a dose of 10 μg/kg for 4–6 days. Initially (until 2019), CD34^+^ cell counts were measured on day 5. Stem cell collection was performed at ≥10/μl CD34^+^ in peripheral blood (pB) on the same day, with a target yield of ≥4 × 10^6^ CD34^+^ cells/kg body weight. If the CD34^+^ cell count in pB was <10/μl on day 5, plerixafor was administered on the evening of day 5, and the first apheresis session (LPH1) was initiated on day 6 if an adequate increase of CD34^+^ cells was observed. In cases of a persistent CD34^+^ count <10/μl on day 6 despite plerixafor, the mobilization attempt was stopped without initiation of an apheresis session, and a second, chemotherapy‐based mobilization attempt was scheduled at a later time point.

From 2019 onward, the local standard practice was modified to better anticipate potential poor mobilizers and enable an early use of plerixafor. All patients were therefore assessed at the apheresis center on day 4, and CD34^+^ counts were measured. Patients with CD34^+^ counts <20/μl received a single dose of plerixafor (20–24 mg s.c.) on the evening of day 4 after individual case discussion. In cases with CD34^+^ counts <5/μl on day 4, the mobilization attempt was terminated and switched to a chemotherapy‐based second mobilization course.

### 2.3 Salvage chemotherapy‐based mobilization in poor mobilizers

A rescue chemotherapy‐based mobilization course was performed in 7 (5%) patients, including four patients in the VCd group and three patients in the Dara‐VTd group. In one patient with an insufficient initial mobilization attempt, the decision was made not to proceed to ASCT, and therefore no second mobilization attempt was performed.

For chemotherapy‐based stem cell mobilization, a cyclophosphamide‐based regimen (usually 1–4 g/m^2^) in combination with G‐CSF was used. G‐CSF (5 μg/kg, twice daily) was typically started 6 days after cyclophosphamide administration. The CD34+ concentration in pB was measured daily, beginning 24 h after white blood cell (WBC) recovery, defined as the first day with WBC >1000/μL. Collections were planned when CD34+ concentration in pB reached ≥10/μL.

### 2.4 Stem cell apheresis

All stem cell collections were performed using the Spectra Optia apheresis system (Terumo BCT) with the continuous MNC protocol (version 11). The target processed blood volume was set to four‐ to five‐fold of the calculated total blood volume. In accordance with the national guidelines, the duration of apheresis was limited to a maximum of five hours.^19^

### 2.5 Evaluation of graft composition

CD34^+^ cell counts in pB and in grafts were determined using single‐platform flow cytometry (BD FACSCanto II, BD Biosciences, Heidelberg, Germany) with four‐color immunophenotyping employing the CD34‐PE/CD45‐FITC reagent (clone 8G12/2D1, BD Biosciences, Heidelberg, Germany) in accordance with previously published guidelines.^20^

The target yield was ≥4 × 10^6^ CD34^+^ cells/kg body weight. Release criteria included a minimum of ≥2 × 10^6^ CD34^+^ cells/kg, negative sterility testing, ≥1 CFU‐GM per 10^5^ cells, and ≥50% post‐thaw viability.

### 2.6 High‐dose chemotherapy and ASCT

All patients received high‐dose melphalan (200 mg/m^2^) as conditioning prior to ASCT, as previously described.

Neutrophil engraftment was defined as the first day of two consecutive days with an absolute neutrophil count >500/μL following stem cell infusion. Platelet engraftment was defined as the first of three consecutive days with a platelet count >20,000/μL in the absence of platelet transfusions. From 2020 onward, all patients received one dose of pegylated G‐CSF after ASCT to ensure timely neutrophil engraftment.

### 2.7 Statistical analyses

All analyses were performed using R version 3.6.2. Descriptive statistics are reported as absolute numbers and percentages, medians (range) or counts (%). Group comparisons were conducted using the Kruskal–Wallis and Chi‐square tests. Multivariable linear and logistic regression models were used to assess factors associated with CD34^+^ mobilization and collected CD34^+^ yield. Statistical significance was set at *p* < .05.

## 3 RESULTS

### 3.1 Patient characteristics

The study included a total of 153 patients with newly diagnosed MM. Of these, 85 patients (56%) received Dara‐VTd and 68 patients (44%) VCd as induction therapy. The median age at apheresis was 62 years (interquartile range [IQR] 56–66) with no significant differences between groups. Other patient characteristics and disease features, including type of myeloma, International Staging System (R‐ISS) stage, and bone marrow plasma cell infiltration, were comparable across groups (Table 1). Patients receiving VCd had a higher number of induction cycles prior to apheresis compared to those receiving Dara‐VTd (4 vs. 3 cycles; *p* < .01). Despite fewer induction cycles, Dara‐VTd was associated with a significantly deeper response prior to apheresis: The rate of ≥VGPR according to IMWG criteria^21^ was 81% in the Dara‐VTd group versus 42% in the VCd group (*p* < .01), even at an earlier stage of induction. No difference was observed in prior radiation therapy before stem cell mobilization between both groups (20% vs. 19%; *p* = .89).

**TABLE 1:** Patient and disease characteristics by induction regime.

		All patients	Dara‐VTd	VCd	
		*n* = 153	*n* = 85 (56%)	*n* = 68 (44%)	
Age in years	Median	62	61	63	*p* = .17
	IQR	56–66	55–66	58–66	
>60 years	*n* (%)	92 (60%)	49 (58%)	43 (63%)	*p* = .48
Sex					
Female	*n* (%)	67 (44%)	36 (42%)	31 (46%)	*p* = .69
Multiple Myeloma subtype					
IgG	*n* (%)	77 (50%)	42 (49%)	35 (52%)	*p* = .53
IgA	*n* (%)	31 (20%)	17 (20%)	14 (21%)	
Light chain	*n* (%)	39 (26%)	24 (28%)	15 (22%)	
Other	*n* (%)	6 (4%)	2 (3%)	4 (5%)	
R‐ISS stage					
Miss	*n*	12	3	9	
I	*n* (%)	36 (26%)	20 (24%)	16 (27%)	*p* = .45
II	*n* (%)	61 (43%)	33 (40%)	28 (48%)	
III	*n* (%)	44 (31%)	29 (35%)	15 (25%)	
Cytogenetics					
Miss	*n*	13	2	11	
Standard risk	*n* (%)	84 (60%)	48 (58%)	36 (63%)	*p* = .53
High risk^a^	*n* (%)	56 (40%)	35 (42%)	21 (37%)	
Bone marrow plasma cell infiltration (%) at diagnosis					
Miss	*n*	9	3	6	
	Median	60.0	60.0	58.0	*p* = .68
	IQR	40.0–72.8	40.0–75.0	40.0–70.0	
Number of induction cycles prior to apheresis	Median	4	3	4	*p* < .01
	IQR	3–4	2–4	4–4	
Number of induction cycles total	Median	4	4	4	*p* = .16
	IQR	4–4	4–4	4–4	
Remission prior to apheresis					
Miss	*n*	1	0	1	
≥VGPR	*n* (%)	97 (64%)	69 (81%)	28 (42%)	*p* < .01
<VGPR	*n* (%)	52 (36%)	16 (19%)	39 (58%)	
Prior radiation therapy	*n* (%)	30 (20%)	17 (20%)	13 (19%)	*p* = .89

Abbreviations: IQR, interquartile range; R‐ISS, Revised International Staging System; VGPR, very good partial remission.

^a^ High‐risk cytogenetics defined as the presence of one or more of the following aberrations detected by FISH: del(17p), t(4;14), t(14;16), gain/amp1q21.

### 3.2 Peripheral blood stem cell (PBSC) mobilization

**FIGURE 1:**
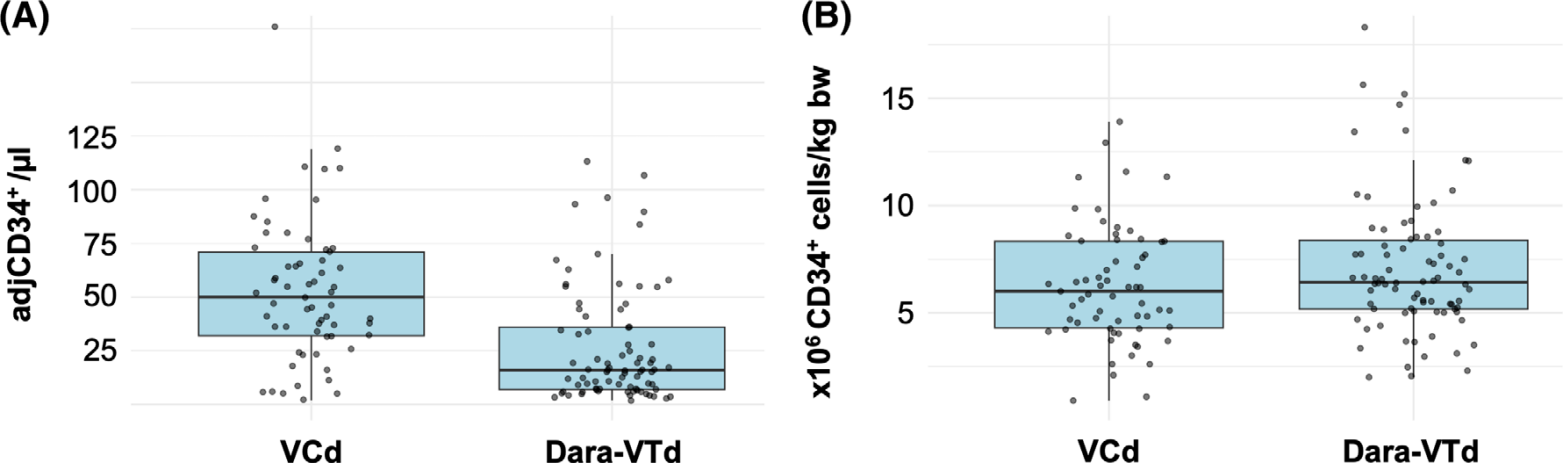
CD34^+^ measurements by treatment group. (A) Adjusted CD34^+^ count in peripheral blood (cells/μl) prior to LPH1. AdjCD34^+^ was measured before any plerixafor administration and reflects intrinsic mobilization capacity; measurement day differed over time (D5 before 2019, D4 from 2019 onward); (B) Total CD34^+^ cells (×10^6^/kg bw) in stem cell grafts after all apheresis sessions. Data shown separately for VCd and Dara‐VTd.

On the day of the LPH1, the median WBC did not differ significantly between the Dara‐VTd and VCd groups (51.0 vs. 47.5 Gpt/L; *p* = .32). Similarly, the median CD34^+^ cell count prior to LPH1 was comparable in both groups (63.0 vs. 54.5/μl; *p* = .11). Plerixafor was used significantly more often in the Dara‐VTd group, with 65% of patients receiving one dose compared to 15% in the VCd group (*p* < .01). To account for plerixafor use, we defined adjCD34^+^ as the CD34^+^ cell count measured prior to any plerixafor exposure. For patients not receiving plerixafor, adjCD34^+^ corresponds to the CD34^+^ count immediately before apheresis. Due to institutional practice changes over time, these measurements were obtained on different mobilization days (day 5 before 2019, day 4 from 2019 onward). The median adjCD34^+^ count was significantly lower in the Dara‐VTd group compared to VCd patients (16 vs. 50/μl; *p* < .01; Figure 1A). Among patients who received plerixafor, the Dara‐VTd group showed a significantly higher increase in CD34^+^ count (56 vs. 28/μl; *p* < .05). In general, plerixafor was administered more frequently as a preemptive measure in imminent mobilization failure than after collection of an insufficient stem cell yield with the LPH1. LPH1 was initiated in a high proportion of patients in both groups without significant differences (97% vs. 93%; *p* = .29).

### 3.3 Stem cell collection and graft composition

Overall collection outcomes and detailed graft composition are summarized in Table 2. The median cumulative CD34^+^ cell yield was 6.4 × 10^6^ CD34^+^ cells/kg body weight (IQR 4.8–8.3 × 10^6^) after Dara‐VTd and 6.0 × 10^6^ CD34^+^ cells/kg body weight (IQR 4.3–8.3) after VCd induction (*p* = .15; Figure 1B).

**TABLE 2:** PBSC mobilization and collection yields.

		All patients	Dara‐VTd	VCd	
		*n* = 153	*n* = 85 (56%)	*n* = 68 (44%)	
CD34^+^ cells/μl pB on LPH1 day					
Miss	*n*	7	3	4	
	Median	55.5	65.5	52.0	*p* = .12
	IQR	36.0–74.5	36.8–83.0	33.8–68.0	
adjCD34^+^ cells/μl pB on LPH1 day					
Miss	*n*	10	3	7	
	Median	32.0	16.0	50.0	*p* < .01
	IQR	11.0–56.0	7.0–36.0	32.0–71.0	
∆ CD34^+^ cells/μl pB post Plerixafor					
Miss	*n*	3	0	3	
	Median	53.5	56.0	28.0	*p* < .05
	IQR	27.2–66.0	29.0–69.9	15.0–53.5	
pB WBC/μl on LPH1 day					
Miss	*n*	7	3	4	
	Median	48.9	51.0	47.5	*p* = .32
	IQR	40.8–59.7	41.3–63.0	39.9–59.3	
Total cells collected at LPH1	×10^8^				
Miss	*n*	8	3	5	
	Median	492.0	514.0	465.0	*p* = 0.20
	IQR	368.0–658.0	368.0–697.5	366.5–579.0	
CD34^+^ cells collected at LPH1	× 10^6^/kg bw				
Miss	*n*	8	3	5	
	Median	6.3	6.4	6.0	*p* = .15
	IQR	4.8–8.3	5.2–8.4	4.3–8.3	
Cumulative CD34^+^ cell yield	× 10^6^/kg bw				
Miss	*n*	8	3	5	
	Median	6.4	6.5	6.2	*p* = .20
	IQR	5.1–8.3	5.3–8.4	4.8–8.3	
Target yield (≥ 4 × 10^6^ CD34^+^ cells/kg bw) achieved with LPH1	*n* (%)	124 (81%)	71 (84%)	53 (78%)	*p* = .38
Target cumulative yield (≥ 4 × 10^6^ CD34^+^ cells/kg bw) achieved total	*n* (%)	133 (87%)	74 (87%)	59 (87%)	*p* = .96
≥ 1 Graft (≥ 2 × 10^6^ CD34^+^ cells/kg bw) achieved with LPH1	*n* (%)	143 (94%)	82 (97%)	61 (90%)	*p* = .09
≥ 1 Graft (≥ 2 × 10^6^ CD34^+^ cells/kg bw) achieved total	*n* (%)	145 (93%)	82 (97%)	63 (93%)	*p* = .43
LPH1 initiated	*n* (%)	145 (95%)	82 (97%)	63 (93%)	*p* = .29
LPH2 initiated	*n* (%)	11 (7%)	3 (4%)	8 (12%)	*p* = .13
Primary mobilization failure	*n* (%)	8 (5%)	3 (4%)	5 (7%)	*p* = .49
Plerixafor use					
Prior 1st apheresis	*n* (%)	65 (43%)	54 (64%)	6 (9%)	*p* < .01
Prior 2nd apheresis	*n* (%)	6 (4%)	2 (2%)	4 (6%)	*p* = .41

Abbreviations: Bw, body weight; LPH1, first apheresis session; LPH2, second apheresis session; pB, peripheral blood; WBC, white blood cells.

In total, 71/82 (86%) patients who underwent Dara‐VTd induction and proceeded to apheresis achieved the target yield (≥4 × 10^6^ CD34^+^ cells/kg body weight) after a single apheresis session. In three (4%) patients, a second apheresis was performed on the following day, and all of them reached the cumulative target yield after two sessions. The remaining eight (10%) patients did not undergo a second apheresis based on individual clinical decisions. Overall, 74/82 (90%) patients who initiated apheresis following Dara‐VTd reached the target yield with their initial mobilization regimen. All 82 patients collected an adequate graft for at least one ASCT (≥2 × 10^6^ CD34^+^ cells/kg body weight). In comparison, 53/63 (84%) patients who received VCd induction achieved the target yield after the LPH1. A second apheresis was performed in eight patients (13%), of whom six reached the target yield thereafter. Overall, 61/63 patients (94%) achieved the anticipated target yield across all apheresis sessions after steady‐state mobilization. Similar to the Dara‐VTd cohort, all 63 patients in whom a stem cell collection was initiated successfully collected a sufficient stem cell graft for at least one ASCT.

### 3.4 Poor mobilizer with a second stem cell mobilization attempt

Overall, eight patients (three after Dara‐VTd and 5 after VCd induction) were unable to proceed to LPH1 due to insufficient CD34^+^ counts (<5/μl in pB) on the day of the planned LPH, despite adequate G‐CSF stimulation. Baseline characteristics of these patients were comparable to the overall cohort (Table S1).

A second, chemotherapy‐based mobilization attempt was performed in seven patients. One patient in the VCd group did not undergo a second mobilization due to an individual decision to switch to a non‐transplant treatment approach. In all seven patients, adequate CD34^+^ counts were measured prior to apheresis (median 40/μl, IQR 27–67/μl), and the stem cell collection was initiated. One patient required an additional dose of Plerixafor. The median stem cell yield was 5.9 × 10^6^ CD34^+^ cells/kg body weight. A single apheresis session was sufficient in six patients; one patient required a second apheresis session. Following this second mobilization attempt, 6/7 (86%) patients reached the target yield. All patients collected at least one stem cell graft containing ≥2 × 10^6^ CD34^+^ cells/kg body weight. After ASCT, comparable engraftment kinetics were observed. No statistically significant differences were observed between the initial induction regimens in these poor mobilizers (Tables S2 and S3).

### 3.5 Factors influencing stem cell mobilization and apheresis yield

A multivariate analysis was performed to assess the potential influence of various factors on the mobilization and collection capacity. Outcome variables were the adjCD34^+^ count prior to LPH1 in peripheral blood (≥20/μl vs. <20/μl) and the achievement of the target collection goal after LPH1 (≥4 vs. <4 × 10^6^ CD34^+^ cells/kg body weight). Prior radiation (odds ratio [OR] 0.2; CI 0.1–0.7; *p* < .05), a high percentage of bone marrow plasma cell infiltration at initial diagnosis (OR 0.3; CI 0.1–0.8; *p* < .05), and use of Dara‐VTd as induction regimen (OR 0.1; CI 0.0–0.2; *p* < .01) were associated with a negative impact on the mobilization capacity prior to LPH1. Age, R‐ISS stage, the presence of high‐risk cytogenetics, and the number of induction cycles before LPH1 did not significantly influence mobilization. Additionally, no significant effects of the tested factors on CD34^+^ collection yield were observed (Table 3).

**TABLE 3:** Multivariable logistic regression model for the mobilization capacity and apheresis yield.

		adjCD34^+^ prior‐LPH1 (≥ 20 vs. < 20/μl)	CD34^+^ yield post‐LPH1 (≥ 4 vs. < 4 × 10^6^ CD34^+^ /kg)
	Reference	OR (CI, *p*‐value)	OR (CI, *p*‐value)
Induction regimen	VCd	**0.1 (0.0–0.2, <.01)**	1.0 (0.2–3.5, .90)
Sex	Male	1.5 (0.6–3.8, .40)	1.3 (0.4–4.1, .70)
Age	≥60 years	0.6 (0.2–1.5, .30)	1.7 (0.6–5.3, .40)
Radiation	No	**0.2 (0.0–0.6, <.01)**	0.4 (0.1–1.6, .20)
Bone marrow plasma cells	<60%	**0.3 (0.1–0.8, <.05)**	1.1 (0.4–3.5, .90)
R‐ISS Stage	I‐II	0.8 (0.3–2.6, .70)	0.6 (0.2–1.8, .30)
Cytogenetics	Standard risk	1.5 (0.5–4.1, .50)	0.9 (0.3–3.0, .90)
Induction cycles	<4	1.1 (0.4–3.0, .90)	0.4 (0.1–1.4, .20)
Remission	<VGPR	2.2 (0.7–8.2, .20)	0.6 (0.1–2.3, .40)

Abbreviations: R‐ISS, Revised International Staging System stage; VGPR, very good partial remission.

### 3.6 Autologous stem cell transplantation and engraftment

Until October 2025, 151/152 (99%) patients with a collected stem cell graft underwent at least one high‐dose chemotherapy with ASCT. In one patient, the ASCT was canceled due to clinical deterioration. The median transplanted CD34^+^ cell count at first ASCT was 2.9 × 10^6^/kg body weight (IQR 2.4–3.4). Except for two patients, all achieved sufficient platelet engraftment at a median of day 12 (IQR 11–13). Neutrophil engraftment was adequately achieved at a median of day 12 in all patients (IQR 10–13) after the first ASCT. There was a significant difference in neutrophil engraftment between Dara‐VTd and VCd patients (median 10 days [IQR 10–11] vs. 13 days [IQR 13–14]; *p* < .01), but not in platelet engraftment (median 12 days [IQR 11–13] vs. 11 days [IQR 11–12]; *p* = .17). It must be noted that most patients in the Dara‐VTd group received an additional dose of pegylated G‐CSF after ASCT, according to the institutional standard protocol introduced in 2020, which is likely to confound the observed differences in neutrophil engraftment.

**FIGURE 2:**
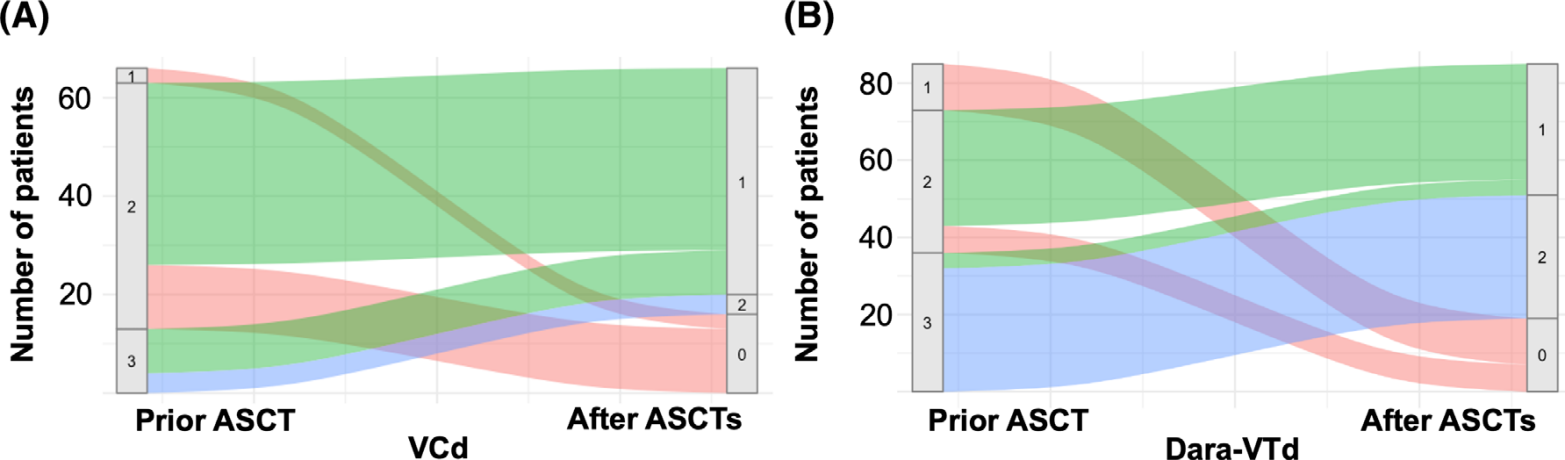
Sankey plots of available stem cell grafts. The plots show the number of collected grafts per patient prior to ASCT (gray bar) and their utilization after the last ASCT until the end of follow‐up (median 4.6 years), including the proportion of grafts not yet infused. (A) VCd; (B) Dara‐VTd.

During follow‐up, 33 patients received a second ASCT (tandem ASCT in 32 patients; one patient received a salvage ASCT). After a median follow‐up of 4.6 years, 152 of 336 collected stem cell grafts (45%) had not been used, including 98 of 194 grafts (51%) in the Dara‐VTd group and 54 of 142 grafts (38%) in the VCd group. Overall, 66 of 85 patients (78%) in the Dara‐VTd group and 50 of 66 transplanted patients (76%) in the VCd group still had at least one graft available after completion of their last ASCT. These data highlight that a substantial proportion of collected grafts remain unused, which contributes to additional costs for mobilization, apheresis, and long‐term storage, and may also impact apheresis unit capacity (Figure 2).

## 4 DISCUSSION

Here we compared stem cell mobilization kinetics and collection efficacy in patients undergoing steady‐state mobilization following two different induction regimens (Dara‐VTd vs. VCd) in a large cohort of newly diagnosed patients with MM.

In the pivotal phase III CASSIOPEIA trial, the use of Dara‐VTd versus VTd in induction treatment was reported earlier. Dara‐VTd showed a significant increase in MRD‐negativity rates and long‐term disease control over VTd.^3^ Therefore, this regimen, as the new standard induction treatment for MM, has paved the way for CD38‐based quadruplet induction therapy in Europe over the past few years. In addition, data for stem cell collection were also reported, showing an impaired median collection yield after CD38‐based induction with 6.7 × 10^6^ CD34^+^ cells/kg body weight compared to 10.0 × 10^6^ CD34^+^ cells/kg body weight in the VTd arm.^22^ Nevertheless, in contrast to our analysis, all patients in this trial underwent a chemotherapy‐based mobilization strategy with cyclophosphamide and G‐CSF, which is generally known to be more effective than steady‐state mobilization.^6^

In our cohort, a steady‐state mobilization approach was used in all patients, raising the question of whether this strategy is sufficient to ensure adequate stem cell collection in newly diagnosed MM patients undergoing a CD38‐containing induction regimen. We did not observe differences in the total collected stem cell yield after both induction regimens, with a total stem cell yield of 6.4 × 10^6^ versus 6.0 × 10^6^ CD34^+^ cells/kg body weight in Dara‐VTd compared to VCd patients. Consequently, the anticipated collection goal of ≥4 × 10^6^ CD34^+^ cells/kg body weight was achieved in the vast majority of patients (90% for Dara‐VTd vs. 94% for VCd), with all patients having collected sufficient cells for at least one ASCT. Nevertheless, when comparing the mobilization kinetics, we observed a significantly lower mobilization efficacy after G‐CSF mobilization in Dara‐VTd patients, which might be partly explained by a possible negative influence of CD38 antibodies on the stem cell mobilization capacity, as suggested earlier.^14^, ^15^, ^18^ Additionally, the use of IMiDs has also shown a negative impact in some studies, which might also contribute to the impaired mobilization efficacy in these patients.^23^, ^24^, ^25^ In our cohort, all patients receiving Dara‐VTd were exposed to thalidomide as part of the induction therapy, whereas patients in the VCd cohort had no prior IMiD exposure. Therefore, the additional use of IMiDs in this cohort might also negatively affect stem cell mobilization. Furthermore, multiple other factors with a possible influence on the mobilization effort have been reported, including prior radiation and high tumor burden at initial diagnosis.^9^, ^26^ In multivariate analysis, we also detected prior radiation, a high tumor burden at initial diagnosis, and the use of Dara‐VTd induction as negative factors on the mobilization capacity, whereas age, R‐ISS stage, number of induction cycles before LPH1, and high‐risk cytogenetics did not significantly affect mobilization.

The addition of plerixafor as a preemptive or rescue strategy in patients with impaired mobilization results has been shown to be an effective method to overcome poor mobilization.^6^, ^7^, ^8^ Likewise, in our study, we found a significant increase in plerixafor use in the Dara‐VTd cohort compared to VCd patients (64% vs. 15%), resulting in the previously reported collection yields. Multiple trials investigating different quadruplet induction regimens incorporating CD38 antibodies and IMiDs have reported similar findings, showing an increased need for plerixafor in these patients regardless of the mobilization strategy used, thereby supporting this approach.^3^, ^4^, ^5^, ^18^, ^27^

Additionally, when accounting for potential factors influencing collection outcomes in a multivariate analysis of our cohort, none of the tested parameters showed a significant effect. This highlights that a structured on‐demand plerixafor strategy can effectively overcome impaired mobilization capacity, even in patients with known risk factors for poor stem cell mobilization.

A limitation of our analysis, beside the retrospective design of the study, is the temporal change in the mobilization algorithm, which resulted in adjCD34^+^ values being measured on different mobilization days. This parameter was deliberately defined to reflect the intrinsic mobilization capacity prior to any plerixafor exposure, rather than absolute CD34^+^ counts on a fixed day, and all key clinical endpoints—including initiation of apheresis, cumulative CD34^+^ yield, and achievement of target collection goals—remained comparable across induction regimens.

The commonly accepted collection goal for hematopoietic stem cell transplantation in MM is 4–6 × 10^6^ CD34^+^ cells/kg body weight. This usually allows for two or even three ASCTs. With the implementation of highly effective treatment options in newly diagnosed MM, it is expected that the use of a second or even third autologous transplantation will decrease significantly in the coming years. Currently, the benefit of tandem transplantation, even in formerly high‐risk myeloma patients (e.g., R‐ISS III, presence of t(4;14) or t(14;16)), is uncertain, as many patients achieve deep responses, including high rates of MRD‐negativity, with a quadruplet induction and consolidation regimen and one autologous transplantation, and also have the option for intensified maintenance treatment.^5^, ^28^, ^29^

Moreover, with the broader availability of effective salvage treatments including immunotherapeutic options such as CAR‐T cells or bispecific antibodies, the use of salvage transplantation is expected to decline significantly.^30^, ^31^, ^32^ In the large phase III ReLapse trial, salvage ASCT was not superior to conventional salvage therapies.^33^ Furthermore, even the use of a collected stem cell graft to restore hematopoietic function in patients after modern immunotherapies with persistent and profound cytopenia, for example, following CAR‐T‐cell therapy, is relatively rare in MM.^34^, ^35^

Our data showed that a significant number of stem cell grafts still have not been used since their collection, despite a median follow‐up period of 4.6 years in the overall patient cohort. Approximately 50% of all collected stem cell grafts have not been infused at the time of data cut‐off. This results in substantial—and often unnecessary—costs for mobilization, apheresis and annual storage.^36^, ^37^, ^38^ Additionally, capacity restrictions must also be considered. Therefore, from an economic point of view, it should be discussed whether generally accepting a lower collection target goal for ASCT in myeloma patients would be reasonable. In the era of highly effective induction regimens, declining use of tandem and salvage ASCT, and increasing availability of novel therapies such as CAR‐T cells and bispecific antibodies, these findings raise important clinical and economic questions. Future prospective and health‐economic studies are warranted to reevaluate optimal collection targets and long‐term storage strategies for autologous stem cell grafts. To our knowledge, long‐term real‐world stem cell graft utilization has not been systematically addressed in prior clinical trials investigating daratumumab‐based induction and stem cell mobilization.

In summary, this large real‐world analysis confirms impaired mobilization after CD38‐based antibody induction, while demonstrating that structured on‐demand plerixafor use remains a feasible and effective strategy to overcome these limitations in routine practice.

## AUTHOR CONTRIBUTIONS

MAR and RT wrote the manuscript. MAR, AS, KZ, MB, KH, and RT collected the data; MAR performed statistical analyses; all authors read and approved the final manuscript and provided administrational support.

## FUNDING INFORMATION

Maximilian Alexander Röhnert is supported by the BMFTR (TRACK, #01KD25030). Malte von Bonin is supported by CAMINO, the Advanced Clinician Scientist Program of TU Dresden, funded by the BMFTR.

## CONFLICT OF INTEREST STATEMENT

The authors have disclosed no conflicts of interest.

## Supporting information


Data S1

## Data Availability

The data that support the findings of this study are available from the corresponding author upon reasonable request.
