# Supplementary material for: Steady‐state mobilization with on‐demand plerixafor after CD38 antibody‐based induction in multiple myeloma patients
Source: Transfusion. 2026 Mar 20;66(4):751–62. doi: 10.1111/trf.70165 (PMC13049232; doi:10.1111/trf.70165)
Supplement: Supplementary file 1 — Data S1 [file TRF-66-751-s001.docx]

**Supplementary material**

Supplementary Table 1

|  |  | **All patients** |  | **Dara-VTd** | **VCd** |  |  |
| --- | --- | --- | --- | --- | --- | --- | --- |
|  |  | n=8 |  | n=3 (38%) | n=5 (62%) |  |  |
| **Age in years** | Median | 65 |  | 64 | 65 |  | p=0.65 |
|  | IQR | 63-66 |  | 63-65 | 65-66 |  |  |
| >60 years | n (%) | 7 (88%) |  | 3 (100%) | 4 (80%) |  | p=0.41 |
| **Sex** |  |  |  |  |  |  |  |
| Female | n (%) | 4 (50%) |  | 2 (67%) | 2 (40%) |  | p=0.47 |
| **Multiple Myeloma subtype** |  |  |  |  |  |  |  |
| IgG | n (%) | 2 (25%) |  | 1 (33%) | 1 (20%) |  | p=0.77 |
| IgA | n (%) | 2 (25%) |  | 1 (33%) | 1 (20%) |  |  |
| Light chain | n (%) | 4 (50%) |  | 1 (34%) | 3 (60%) |  |  |
| **ISS Stage** |  |  |  |  |  |  |  |
| Miss | n | 1 |  | 0 | 1 |  |  |
| I | n (%) | 2 (29%) |  | 0 (0%) | 2 (50%) |  | p=0.23 |
| II | n (%) | 1 (14%) |  | 1 (33%) | 0 (0%) |  |  |
| III | n (%) | 4 (57%) |  | 2 (67%) | 2 (50%) |  |  |
| **Cytogenetics** |  |  |  |  |  |  |  |
| Miss | n | 1 |  | 0 | 1 |  |  |
| Standard-risk | n (%) | 4 (57%) |  | 0 (0%) | 4 (100%) |  | p=0.01 |
| High-risk^#^ | n (%) | 3 (43%) |  | 3 (100%) | 0 (0%) |  |  |
| **Bone marrow plasma cell infiltration (%) at diagnosis** |  |  |  |  |  |  |  |
|  | Median | 60.0 |  | 70.0 | 60.0 |  | p=0.34 |
|  | IQR | 60.0-72.5 |  | 65.0-75.0 | 60.0-60.0 |  |  |
| **Remission prior to apheresis** |  |  |  |  |  |  |  |
| ≥VGPR | n (%) | 5 (63%) |  | 2 (67%) | 3 (60%) |  | p=0.85 |
| <VGPR | n (%) | 3 (37%) |  | 1 (33%) | 2 (40%) |  |  |
| **Prior radiation therapy** | n (%) | 1 (13%) |  | 0 (0%) | 1 (20%) |  | p=0.41 |

Supplementary Table 1: Patient and disease characteristics by induction regime of poor mobilizers

*IQR* interquartile range, *ISS* International Staging System, *VGPR* Very Good Partial Remission

^#^ High-risk cytogenetics defined as the presence of one or more of the following aberrations detected by FISH: del(17p), t(4;14), t(14;16), gain/amp1q21

Supplementary Table 2

|  |  | **All patients** |  | **Dara-VTd** | **VCd** |  |  |
| --- | --- | --- | --- | --- | --- | --- | --- |
|  |  | n=8 |  | n=3 (38%) | n=5 (62%) |  |  |
| **First mobilization attempt (Steady State)** | | | | | | | |
| **pB CD34^+^ cells/μl max after steady-state** |  |  |  |  |  |  |  |
| Miss | n | 0 |  | 0 | 0 |  |  |
|  | Median | 2 |  | 2 | 3 |  | p=0.57 |
|  | IQR | 1-4 |  | 1-4 | 1-4 |  |  |
| **pB WBCmax in Gpt/L after steady-state** |  |  |  |  |  |  |  |
| Miss | n | 2 |  | 0 | 2 |  |  |
|  | Median | 66.0 |  | 32.5 | 41.0 |  | p=1.0 |
|  | IQR | 14.0-60.0 |  | 8.0-70.0 | 20.0-61.9 |  |  |

Supplementary Table 2: Characteristics of the first mobilization attempt in poor mobilizers

Supplementary Table 3

|  |  | **All patients** |  | **Dara-VTd** | **VCd** |  |  |
| --- | --- | --- | --- | --- | --- | --- | --- |
|  |  | n=7 |  | n=3 (42%) | n=4 (58%) |  |  |
| **Second mobilization attempt (chemotherapy-based)** | | | | | | | |
| **Days from start mobilization to LPH1** |  |  |  |  |  |  |  |
| Miss | n | 0 |  | 0 | 0 |  |  |
|  | Median | 14 |  | 15 | 14 |  | p=0.49 |
|  | IQR | 14-15 |  | 14-15 | 14-15 |  |  |
| **CD34^+^ cells/μl pB on LPH1 day** |  |  |  |  |  |  |  |
| Miss | n | 0 |  | 0 | 0 |  |  |
|  | Median | 40.0 |  | 67.0 | 35.0 |  | p=0.48 |
|  | IQR | 58.5-57.5 |  | 46.0-110.5 | 29.2-42.0 |  |  |
| **pB WBC/μl on LPH1 day** |  |  |  |  |  |  |  |
| Miss | n | 0 |  | 0 | 0 |  |  |
|  | Median | 11.1 |  | 11.1 | 11.3 |  | p=0.72 |
|  | IQR | 10.4-12.2 |  | 10.6-13.5 | 9.2-12.1 |  |  |
| **Total cells collected at LPH1** | **x10^8^** |  |  |  |  |  |  |
| Miss | n | 0 |  | 0 | 0 |  |  |
|  | Median | 436.0 |  | 408.0 | 455.0 |  | p=1.00 |
|  | IQR | 348.4-526.0 |  | 348.4-609.4 | 384.8-500.0 |  |  |
| **CD34^+^ cells collected at LPH1** | **x10^6^/kg bw** |  |  |  |  |  |  |
| Miss | n | 0 |  | 0 | 0 |  |  |
|  | Median | 5.9 |  | 3.9 | 6.0 |  | p=1.00 |
|  | IQR | 3.9-10.5 |  | 3.9-7.2 | 5.2-6.1 |  |  |
| **Cumulative CD34^+^ cell yield** | **x10^6^/kg bw** |  |  |  |  |  |  |
| Miss | n | 0 |  | 0 | 0 |  |  |
|  | Median | 5.9 |  | 3.9 | 6.0 |  | p=0.48 |
|  | IQR | 4.7-6.2 |  | 3.9-7.2 | 5.8-6.1 |  |  |
| **Target yield (≥ 4x10^6^ CD34^+^ cells/kg bw) achieved with LPH1** | n (%) | 4 (57%) |  | 3 (75%) | 1 (33%) |  | p=0.27 |
| **Target cumulative yield (≥ 4x10^6^ CD34^+^ cells/kg bw) achieved total** | n (%) | 5 (71%) |  | 4 (100%) | 1 (33%) |  | p=0.05 |
| **≥ 1 Graft (≥ 2x10^6^ CD34^+^ cells/kg bw) achieved with LPH1** | n (%) | 7 (100%) |  | 4 (100%) | 3 (100%) |  | p=1.0 |
| **LPH1 initiated** | n (%) | 7 (100%) |  | 4 (100%) | 3 (100%) |  | p=1.0 |
| **LPH2 initiated** | n (%) | 1 (14%) |  | 1 (25%) | 0 (0%) |  | p=0.35 |
| **Plerixafor use** | n (%) | 1 (14%) |  | 1 (25%) | 0 (0%) |  | p=0.46 |

Supplementary Table 3: Characteristics of the second mobilization attempt (chemotherapy-based) in poor mobilizers

*bw* body weight, *pB* peripheral blood, *WBC* white blood cells, *LPH1* first apheresis session, *LPH2* second apheresis session
